# Strong confinement-induced engineering of the *g* factor and lifetime of conduction electron spins in Ge quantum wells

**DOI:** 10.1038/ncomms13886

**Published:** 2016-12-21

**Authors:** Anna Giorgioni, Stefano Paleari, Stefano Cecchi, Elisa Vitiello, Emanuele Grilli, Giovanni Isella, Wolfgang Jantsch, Marco Fanciulli, Fabio Pezzoli

**Affiliations:** 1 LNESS and Dipartimento di Scienza dei Materiali, Università di Milano Bicocca, via Cozzi 55, Milano 20125, Italy; 2 Dipartimento di Scienza dei Materiali, Università di Milano Bicocca, via Cozzi 55, Milano 20125, Italy; 3 LNESS and Dipartimento di Fisica, Politecnico di Milano, via Anzani 42, Como 22100, Italy; 4 Institut für Halbleiter-und Festkörperphysik, Johannes Kepler University, Altenbergerstrasse 69, Linz 4040, Austria

## Abstract

Control of electron spin coherence via external fields is fundamental in spintronics. Its implementation demands a host material that accommodates the desirable but contrasting requirements of spin robustness against relaxation mechanisms and sizeable coupling between spin and orbital motion of the carriers. Here, we focus on Ge, which is a prominent candidate for shuttling spin quantum bits into the mainstream Si electronics. So far, however, the intrinsic spin-dependent phenomena of free electrons in conventional Ge/Si heterojunctions have proved to be elusive because of epitaxy constraints and an unfavourable band alignment. We overcome these fundamental limitations by investigating a two-dimensional electron gas in quantum wells of pure Ge grown on Si. These epitaxial systems demonstrate exceptionally long spin lifetimes. In particular, by fine-tuning quantum confinement we demonstrate that the electron Landé *g* factor can be engineered in our CMOS-compatible architecture over a range previously inaccessible for Si spintronics.

## Abstract (web summary)

Ge/Si heterojunctions are prominent candidates for spintronics, but the spin-dependent phenomena have been elusive. Here, Giorgioni *et al*. report long spin relaxation and coherence times in a two dimensional electron gas confined in quantum wells of pure Ge grown on SiGe-buffered Si substrates.

Spin–orbit interaction (SOI) couples the quasi-momentum of charged particles to their spin^1^. This effect has sparked considerable interest because it results in a suitable spin splitting even in the absence of external magnetic fields. SOI governs spin-dependent phenomena such as Rashba physics^2^,^3^,^4^,^5^,^6^, persistent spin helix states^7^,^8^,^9^, spin Hall^10^,^11^,^12^ and spin Seebeck effects^13^,^14^, offering novel and exciting perspectives for utilizing spin currents in non-magnetic materials^15^. This holds the promise for the end-of-the-roadmap implementation of semiconductor spintronics^16^.

Elemental group IV semiconductors, which are the fundamental materials of mainstream microelectronics, notably own a centrosymmetric crystal structure^1^,^17^. Although the resulting bulk inversion symmetry seemingly yields a negligible splitting of the spin sublevels, the spatial symmetry and relative order of their energy bands give rise to useful, albeit untapped, spin–orbit coupling (SOC) phenomena. The band-dependence of the spin mixing in the carrier wave functions is of crucial importance in defining contributions to the spin–flip scattering mechanisms and its control, if achieved, is expected to enhance the spin lifetime of the carriers^18^,^19^,^20^. Similarly, the Landé *g* factor is governed by spin-dependent but orbital contributions due to the nonzero off-diagonal matrix elements of momentum that couple the lowest conduction band to remote bands^21^. A successful manipulation of the *g* factor can eventually facilitate the susceptibility of the spin state of the charge carrier to an external field^1^.

In Si, these SOC-dependent phenomena result in an exceptionally long spin lifetime^18^,^22^ but end up in a negligible deviation of the electron *g* factor from the isotropic free carrier value *g*_0_∼2 (ref. 23). Seminal works demonstrating tailoring of the spin properties in Si rather focused on low-dimensional Si/SiGe heterosystems, in which SOI gives rise to a momentum-dependent term in the Hamiltonian becoming more important at the interfaces as a result of the induced spatial inversion asymmetry^24^,^25^,^26^. Yet the *g* factor tunability in such systems remained very small^27^.

In this context, we turned our attention to Ge because it shares with Si the key prerequisites for any practical implementation of quantum information processing, namely a long spin-relaxation time and a substantial abundance of spin-less isotopes^28^,^29^. In addition, by hosting conduction band electrons in the L- rather than X-valleys^20^,^30^, Ge features a highly anisotropic *g* factor^31^. In view of its full compatibility with the technology of integrated circuits and its exceptionally high bulk mobility, Ge also increasingly is seen as a viable option for replacing Si in conventional high-frequency logics^32^ and can thus be regarded as an attractive candidate for transport in novel spintronic architectures.

Recently, intriguing phenomena have been revealed in Ge-based heterostructures. Cubic-**k** terms have been shown to dominate the **k**·**p** SOI Hamiltonian of two-dimensional hole gases^33^. Electric-field-induced tuning of the hole *g* factor^34^ has been reported in hybrid devices made from superconductors and self-assembled nanocrystals^35^, while core–shell Ge/Si nanowires^36^ have been envisioned as hosts for Majorana fermions^37^.

To date, however, efforts have been mainly focused on the spin physics of holes. Besides the large lattice mismatch, which induces growth defects and poor material and interface quality, the spontaneous type II band alignment at Ge/Si heterojunctions^38^,^39^ has so far precluded the experimental study of spin–orbit mechanisms for conduction electrons confined in Ge. Indeed, charge carriers are spatially separated by the built-in potential, which favours holes (electrons) at the Ge (Si) side of the heterointerface.

Here we propose the use of heterostructures based on Ge and demonstrate that a two-dimensional electron gas (2DEG) confined in quantum wells of pure Ge offers a sizeable control over the *g* factor and exhibits long spin relaxation and coherence times, eventually putting forward the potential of Ge in bridging the gap between spintronic concepts and semiconductor device physics.

## Results

### Confinement of conduction electrons in Ge heterostructures

**Figure 1:**
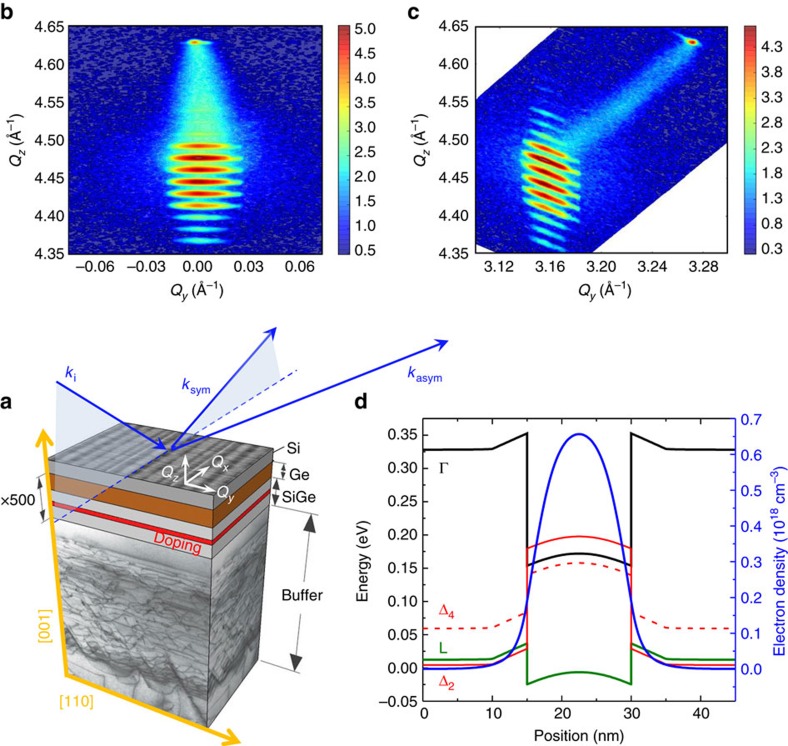
Sample structure and band-edge alignment. (**a**) Sketch of the structure (not to scale) of the n-type (P atoms) modulation doped Ge/SiGe QWs samples. Each sample consists of a 500-fold-stack of QWs grown on (001)Si substrates. A sketch of the incident, **k**_*i*_, and diffraction wave vectors, **k**_sym_ and **k**_asym_, corresponding respectively to the (004) and (224) reciprocal lattice points, is shown. *Q*_*i*_ refers to the measured scattering vector, here *i*=*x*, *y*, *z*. (**b**,**c**) Symmetric (004) and asymmetric (224) XRD reciprocal space maps of the sample with 20 nm QWs, respectively. The colour scale bar represents the XRD logarithmic intensity as counts-per-second. (**d**) Calculated conduction band alignment and electron density in the Ge/SiGe multiple QW structure.

**Figure 2:**
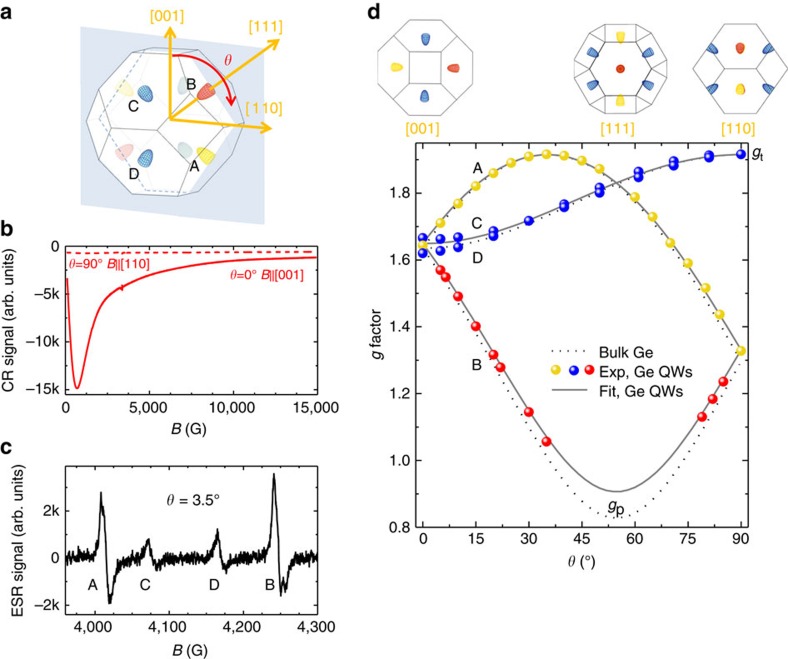
CESR of Ge QWs. (**a**) Brillouin zone of bulk Ge. *θ* is the angle between the [001] crystallographic direction and the magnetic field B, which scans towards the [110] direction. The ellipsoidal isoenergetic surfaces of the conduction band at the L point are also shown. (**b**) CR signal in Ge QWs measured at *T*=2 K for *θ* =0° (solid red line) and *θ* =90° (dashed red line). (**c**) ESR signals from conduction electrons in Ge QWs measured at *T*=2 K and *θ* =3.5°, after subtracting a linear background. (**d**) Values of *g* factor measured from the ESR peaks at *T*=2 K in 20 nm Ge QWs as a function of *θ*. The correspondence between the angle *θ* and the main crystallographic directions are highlighted in the upper part of the figure. Labels from A to D establish the relation of the branches both with the peaks in **c** and with the valleys in **a**. *g*_p_ and *g*_t_ are the lowest and the highest values of *g*, respectively. Upper part: sketches of the Brillouin zone when B is directed along the main crystallographic axis.

In light of the pivotal advances reported in the field of Si photonics^40^,^41^ we expect that band-gap engineering in SiGe alloys will similarly provide advantages to semiconductor spintronics by opening unexplored pathways for the full exploitation of Ge. The degrees of freedom offered by strain and alloying in dictating the band-edge offsets in SiGe heterostructures motivated us to design n-type modulation (n-mod) doped devices on Si consisting of a 500-fold-stack of pure Ge quantum wells (QW) embedded in Ge-rich SiGe barriers having a 10 nm thick phosphorous doped region at their centre (Fig. 1a). The individual layers were engineered in order to obtain a negligible strain with respect to the SiGe buffer, as confirmed by high-resolution x-ray diffraction (HRXRD) measurements (see Fig. 1b,c and the Supplementary Note 1). Such strain-compensation accommodates the compressed QW in between tensile strained barriers and precludes the formation of additional defects at the interfaces. The resulting Ge/SiGe heterojunction allows us to gather direct access to a type I band alignment, with a notable accumulation of L-valley electrons (see Figs 1d and 2a) in the Ge well due to a robust confining potential of the order of 60 meV. This, combined with conduction electron spin resonance (CESR), permits experimental detection of the electron *g* factor theoretically predicted in Ge more than a decade ago^42^. Here we report a systematic study on samples that, according to HRXRD, differ by the QW thickness, namely 20±1, 17±1 and 16±1 nm.

### Electron spin resonance of 2DEG in Ge

At low temperature, we found in CESR a cyclotron resonance (CR) that strongly depends on the relative orientation of an external magnetic field **B** with respect to the sample surface. As shown in Fig. 2b, the sample with the largest width of the QWs and without remote doping does not show a CR signal when **B** lies along the [110] direction (in-plane field). On the other hand, when **B** is rotated towards the [001] growth direction (perpendicular field), the spectrum exhibits a very pronounced, broad signal. This behaviour is a clear signature that carriers are confined in the (001) plane, where they can undergo cyclotron motion driven by the electric field of the microwave^24^. This well-defined CR and its characteristic dependence upon illumination, shown in detail in the Supplementary Figs 1 and 2, provides direct proof of the existence of a 2DEG in the QWs plane^24^ and the absence of low temperature localization of carriers on impurity sites.

As a consequence, we have direct access to the intrinsic spin-dependent properties of conduction electrons. This constitutes a remarkable difference with respect to previous electron spin resonance studies applied to Ge (refs 28, 43, 44, 45, 46, 47). Apart from work focussed on electrons bound to donors^28^,^43^,^44^, very few experiments suggested the peculiar presence of an ESR due to delocalized electrons in antimony-doped bulk Ge at low temperatures^45^,^46^,^47^. Such finding was ascribed to partial population of conduction band states by the built-in inhomogeneous strain fields randomly experienced by electrons at different Sb sites. Instead, our heteroepitaxial n-mod architecture naturally guarantees itinerant electrons in the Ge layer and their concomitant spatial separation from the remote donors that reside in the SiGe barrier.

This point is further corroborated by the following results. In addition to the CR signal, Fig. 2c shows that four well-resolved CESR peaks appear in n-mod samples. These peaks (A–D in Fig. 2c) strongly shift with increasing from 0° to 90° the angle *θ* between **B** and the normal to the sample surface. This dependence demonstrates a highly anisotropic *g* factor, as summarized in Fig. 2d. Notably, we did not succeed in observing peaks corresponding to electrons localized on P donors, neither in the SiGe barriers nor in the Ge wells. This confirms that P atoms in the barriers are ionized and have not sizeably diffused during growth. The origin of the narrow resonance lines shown in Fig. 2c and of their marked angular dispersion can be rationalized as detailed below.

In bulk Ge each conduction band edge at the four equivalent L points of the Brillouin zone has an ellipsoidal energy surface oriented along a 〈111〉 crystal direction (Fig. 2a). According to Roth and Lax^31^, the *g* factor matrix of free electrons reflects such spheroidal shape and its axial *C*_3v_ symmetry^45^. Hence for any angle *ϕ* between the external field and the major axis of one ellipsoid of revolution, the concomitant effective value of *g* can be obtained as follows^48^:


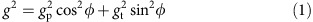


where *g*_p_ and *g*_t_ are the two independent parallel and transverse components lying along or being normal to the major axis of the ellipsoid, respectively. The *g* factor anisotropy, however, can be better appreciated, as in Fig. 2d, by recasting equation (1) with respect to the angle *θ* (see Supplementary Note 2).

Figure 2d compares our experimental data for QWs and the angular dependence of the *g* factor of conduction electrons in bulk Ge as obtained by using the *g*_p_ and *g*_t_ values from refs 47, 49 (dotted lines). Their striking agreement demonstrates, at a glance, that the CESR features of Ge wells originate from itinerant L-valley electrons and that the heterostructures preserve the bulk *C*_3v_ symmetry of the *g*-tensor. Such finding is in sharp contrast to the behaviour of the magneto-conductivity tensor, which rules the CR response (Fig. 2b). This might be a consequence of the fact that the latter is mostly determined by heterointerface properties, while deviations of the *g* factor from the free electron value are caused first of all by SOC (ref. 24).

It is worth noting that the observation of a well-resolved CESR multiplet proves that spin relaxation of conduction electrons in QWs is dominated by zone-centre intravalley rather than zone-edge intervalley electron–phonon coupling^20^. The latter, due to scattering among the different L minima, would have otherwise averaged out the *g* factors, eventually yielding a single CESR line^44^. We emphasize that the inversion symmetry of the Ge lattice is well-known to exclude D'yakonov–Perel type spin–flip processes so that spin relaxation is essentially mediated by the Elliott–Yafet mechanism. This feature and the unique SOC experienced by thermal electrons at the conduction band edge of Ge have been recently suggested resulting in exceptionally long-lived electron spin states^19^,^20^. By working at cryogenic temperatures, we could selectively quench the intervalley scattering, that previous literature work recognized as one of the crucial factors in limiting the experimentally accessible spin-relaxation times^21^,^49^. This will open up the possibility to precisely unveil relevant spin–flip and dephasing mechanisms.

Figure 2d allows us to obtain via equation (1) the *g*_p_ and *g*_t_ values for the QWs (solid lines) and to identify the pristine valleys (A–D in Fig. 2a) giving rise to the observed resonance lines. To better appreciate this, we can start considering *θ*=0° where all the CESR peaks merge at *g*∼1.66. The weak removal of degeneracy, which can be noticed in the QW data of Fig. 2d, is due to ∼0.5° misalignment of the sample towards the [110] direction during the experiments. By increasing *θ* to ∼55°, the external field aligns with the major axis of the ellipsoidal energy surface of valley B, highlighted in red in Fig. 2a, and its associated *g* factor decreases to the minimum value, that is 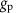. The severe reduction of the CESR intensity with the *g* value prevented us from observing the resonance lines at *g*<1.05 (see Supplementary Fig. 3). For valleys C and D (blue in Fig. 2a) a 90° increase of *θ* yields an increase of *g* from 1.66 up to the largest value, namely *g*_*t*_.

A closer look to Fig. 2d already points out that at a fixed *θ*, the *g* factor of bulk and QWs are different. In particular, the mismatch is maximum when *g*=*g*_p_ and vanishes when *g*=*g*_t_. Those changes can indeed be used as sensitive probes of the electronic band structure as they manifest the combined effects of strain and confinement on SOC (ref. 42) In this work we were able to disentangle these two contributions by focussing entirely on the latter. In fact, while adjusting the confinement via the QW width, all our heterostructures retain the same strain level being set by the lattice mismatch between Ge and the buried SiGe buffer (see also data in Supplementary Note 1).

### Tuning of the electron Landé *g* factor

**Figure 3:**
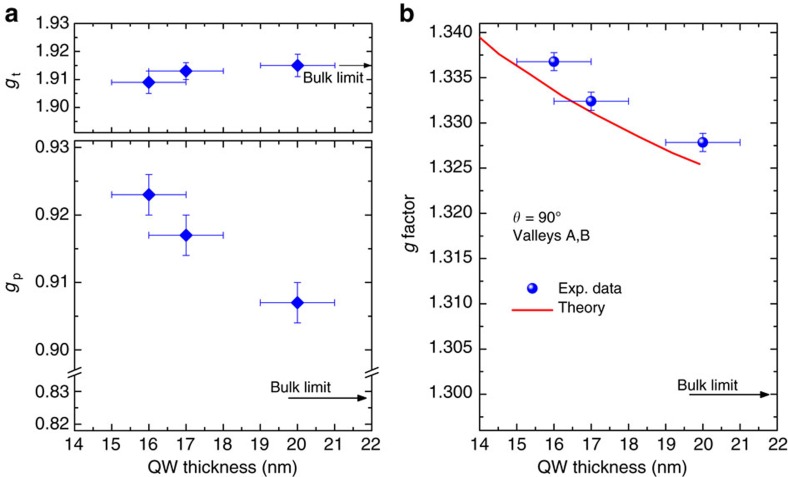
Confinement-induced *g* factor tuning. (**a**) Values of *g*_*p*_ and *g*_*t*_ parameters reported in ref. 47 for bulk Ge (arrows), and obtained in this work (diamonds) for Ge QWs with different thickness. Errors bars were derived from the least square fitting analysis of the experimental data. (**b**) *g* factor values at *θ* =90° for the valleys A and B as a function of the QW thickness. The experimental data for Ge QWs (dots) are reported along with the values calculated by Baron *et al*. in ref. 42 (red line), and the reference values of bulk Ge according to ref. 47. Error bars of the *g* factor were derived from the resonance field uncertainty.

Figure 3a reports *g*_p_ and *g*_t_ as a function of the well thickness (diamonds) along with the corresponding bulk Ge benchmarks (arrows) taken from the literature^47^. Remarkably, while *g*_t_ of bulk and QW coincide within the experimental error, *g*_p_ becomes substantially larger than the bulk limit as the QW width decreases. The findings summarized in Fig. 3 constitute the experimental proof of a puzzling SOC effect induced by interactions between the lowest conduction band at the L point and the other close and remote bands. Such phenomena were unveiled by **k**·**p** perturbation theory by Baron *et al*.^42^, who anticipated the renormalization of the *g* factor of L-valley electrons in Ge/SiGe QWs. The excellent agreement between theory and experiments can also be noticed in Fig. 3b, where our data at *θ*=90° for degenerate A and B valleys (dots) are directly compared with the corresponding calculations from ref. 42 (solid line).

We emphasize that, although the manipulation of the electron *g* factor has been largely addressed in QWs of III–V compounds^50^,^51^, our approach discloses a large shift directly in group IV materials. Here we leverage on the low dimensionality of the structures to extend the electron *g* factor tunability by more than one order of magnitude compared with the experimental works on Si-based systems published to date^27^,^52^,^53^ (See Table 1). The results reported in Fig. 3 also substantiate the possibility suggested in ref. 42 of tuning the *g* factor in Ge QWs by exploiting the additional confinement induced by an external electric field.

CESR investigation of the anisotropic electron dispersion of L-valleys provides insight also into the electron spin coherence. To address this further, we now focus on the CESR lineshape in an attempt to identify the homogeneous Lorentzian linewidth Δ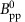 and possible broadening mechanisms of the resonance peaks that might conceal transverse spin-relaxation processes^54^.

### Analysis of CESR linewidth and spin dephasing mechanisms

**Figure 4:**
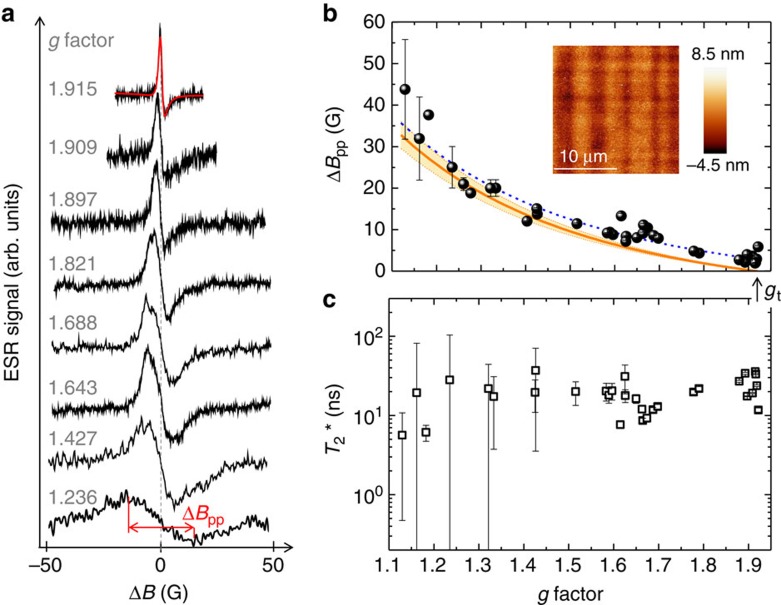
CESR linewidth analysis. (**a**) CESR peaks for valleys A and B of the 20 nm Ge QWs observed at the different *g* factors as a function of Δ*B*=*B*−*B*_res_, where *B*_res_ is the the resonance field. The spectra are vertically shifted for clarity. The peak-to-peak linewidth 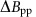 of the ESR line at the smallest *g* factor is shown. The ESR line at *g*=1.915 is reported together with the Lorentzian-based composition of the three contributions (red line) to the ESR signal (see Supplementary Note 5). (**b**) Full dots are the measured 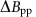 for all the L-valleys as a function of *g*. Error bars were derived from least squares fit of the lineshape of the resonance lines. The orange line is the calculated inhomogeneous broadening 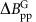 due to the interface roughness in 20 nm Ge QWs. Shadowed area corresponds to the error bar of the calculated 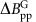, resulting from the roughness error. The dashed blue line corresponds to the orange curve, shifted by 2.9 G. Inset: AFM image of the surface of the sample having 20 nm thick QWs. (**c**) 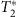 values obtained by the linewidth of the ESR lines, after removing the inhomogeneous 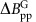 broadening contribution due to the fluctuations of the QW width. Error bars of 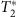 were obtained by taking into account the experimental error of the surface roughness as detailed in the Supplementary Note 3.

Figure 4a reports CESR lines corresponding to various *g* factors measured in the sample with the widest QWs. For a better comparison, the spectra are shifted by an amount equal to their own resonance field. The spectra show linewidths around 1–50 G, at least two orders of magnitude larger than those demonstrated in a single Si QW (ref. 27). This points out that the 500-fold-stack of QWs is a key-enabling factor to enhance the signal, hence giving access to CESR resonances that in Ge would have been otherwise concealed to the observation.

Figure 4a shows that the measured peak-to-peak linewidth 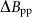 unexpectedly decreases when the *g* factor increases. These peaks belong to either valley A or B. The upper panel of Fig. 4b summarizes similar results also for valleys C and D, thus clarifying that 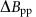 does not depend upon the valley index, but it is exclusively linked to the value of *g*. The observation of a similar broadening for peaks originating from independent valleys further indicates the dominant role played by the intravalley relaxation.

To gather better insight into decoherence mechanisms, we start noticing that the epitaxial growth of strained Ge layers is always accompanied by surface roughness, yielding fluctuations of the QW width. On the time scale of momentum relaxation within a valley, the electrons experience scattering through regions of randomly changing *g* factor, which reflect the in-plane variations of the well thickness. As a consequence, the spin state of the electron dephases providing a relevant source of inhomogeneous Gaussian broadening 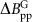 of the CESR peaks. As detailed in the Supplementary Note 3, we evaluated this contribution by using the root-mean-square roughness of the sample surface as obtained by Atomic Force Microscopy (inset of Fig. 4b). The latter is ∼2nm for all the QW samples (see ‘Methods' section). The results of this analysis are displayed as an orange line in Fig. 4b, and highlight two interesting points. First, 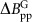 vanishes at high *g* values. In particular, 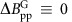 when *g*=*g*_t_, which explains why the measured CESR peaks at *g*_t_ (see an example in Fig. 4a) are the only ones which can be well approximated with a Dysonian-based ESR signal (red line in Fig. 4a). Second, 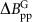 gains weight when *g* decreases. However, the not complete agreement with the experiments evidences that, although interface roughness is the main source of CESR broadening, other contributions have to be taken into account to fully explain the observed lineshapes. In particular, we notice that to a first approximation the experimental data can be recovered by a rigid shift of ∼2.9 G to the calculated 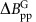 (compare solid and dotted lines in Fig. 4b). Since this offset points towards isotropic decoherence mechanisms, we suggest the following scenario to explain the physics leading to that 2.9 G broadening.

The stochastic nature of the diffusion process washes out the inhomogeneous magnetic fields arising from the nuclear spins of the naturally occurring ^73^Ge isotopes. Hence itinerant L-valley electrons experience an effective suppression of the hyperfine relaxation and are expected to yield the so-called motional narrowing, that is, a reduced CESR linewidth. Nevertheless, during their random walk within the QW plane, mobile electrons are likely to reside for a finite time in smooth potential islands induced by thickness fluctuations before jumping into a neighbouring in-plane site. This partial localization enhances the Fermi contact interaction between the spin and the local nuclear fields, sustaining dephasing and, in turn, CESR broadening. The isotropic component of the linewidth pointed out in Fig. 4b can thus be accounted for by the two aforementioned opposing effects, namely hyperfine coupling and motional narrowing. In our Ge QWs the prominent role of the latter leads to a remnant hyperfine broadening of 2.9 G. This is substantially narrower than the 10 G resonance linewidth of electrons fully bound to shallow donors that is well known for bulk samples with natural isotopic abundance of ^73^Ge (refs 28, 43).

It shall be noted that the phenomenon discussed above neglects broadening due to spin–flip processes, consistently with the spin-relaxation times addressed in the following.

Data in Fig. 4b further show the occurrence of slightly different linewidths at the same *g* value, thus suggesting the presence of additional, albeit weaker, dephasing mechanisms. With this respect, it is illuminating to note that we measured two *g*_*t*_ peaks: one at *θ*∼35° (Fig. 2d), having 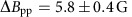, and the other at *θ*=90°, having 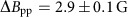. Similar 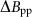 ratios of these two CESR lines have been systematically observed in all the QW samples, thus highlighting that the transverse spin relaxation is slower for an in-plane field, that is, *θ*=90°. This finding can be understood in terms of the Elliott–Yafet mechanims^20^. In a 2DEG the probability of spin-dependent scattering processes is proportional to *σ*·(**k** × **k**′), where *σ* is the electron spin, and **k** and **k**′ are the momenta of the initial and final electron states (see Supplementary Note 4). Scattering events thus provide transverse relaxation of in-plane spin components, namely the ones probed at *θ*=0°, but do not affect out-of-plane spin components. As a consequence, as *θ* decreases towards 0°, the relaxation induced by Elliott–Yafet mechanisms becomes more important, manifesting itself in our experimental data as a sizeable contribution to the broadening of CESR lines.

The observation of larger linewidths at small *θ* values also rules out decoherence due to Rashba SOI. Although precluded by the symmetric design of our n-mod structures, this effect can still possibly occur because of the rotoinversion asymmetry induced by the finite, unavoidable roughness of the interfaces or asymmetric doping^55^. The nature of such SOI, if any, would lead to a Rashba field oriented within the 2DEG plane^3^ and would provide an additional channel of transverse spin relaxation that, as opposed to our findings, increases the linewidth when *θ* approaches 90° (ref. 56).

After having discussed the mechanisms contributing to the observed CESR linewidth, we can determine the relaxation time of the spin ensemble 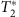, which provides a lower limit for the spin decoherence time *T*_2_ (ref. 54), as follows:


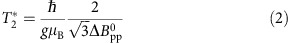


where *ħ* is the reduced Planck constant, *μ*_B_ the Bohr magneton, *g* is obtained from the CESR peak position, and Δ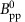 can be obtained by the following relation^57^:


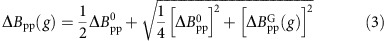


using the measured Δ*B*_pp_(*g*) shown in Fig. 4b, and the inhomogeneous broadenings Δ
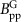
(*g*) as calculated in the Supplementary Note 3.

The values of 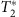 for the widest QW sample are summarized in Fig. 4c. Similar data have been found also for narrower QWs (Supplementary Fig. 4). In agreement with the physical picture of itinerant electrons subject to fluctuating confinement potentials, 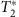 turns out to be about 20 ns, which is about two times longer than the hyperfine-limited dephasing times of electrons bound to shallow donors^28^ and more in line with magneto-optical data for conduction band electrons in bulk Ge (ref. 58). In the latter case the spin decoherence time was found to be anisotropic, reflecting the intervalley scattering regime^49^. Figure 4b demonstrates that when the intravalley relaxation is dominant, the ensemble dephasing time is not *g*-factor-dependent and thus isotropic.

### CESR power dependence and spin-lattice relaxation

In the following, we extract the spin-lattice or longitudinal relaxation time *T*_1_ from the power (*P*) dependence of continuous wave ESR (ref. 54). To this end, we carried out selected measurements in a cylindrical cavity with high Q-factor and a finite electric field of the microwave within the sample (see ‘Methods' section for further details). Moreover, we restricted ourselves to the analysis of CESR lines at *g*=*g*_t_, because, as shown before, those are unaffected by the inhomogeneous broadening induced by the interface roughness.

**Figure 5:**
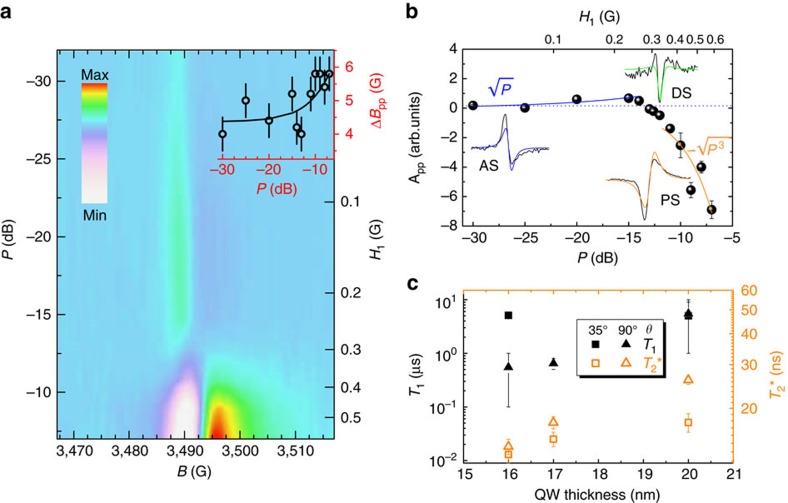
CESR power dependence and spin-relaxation times. (**a**) Colour-coded intensity map of the CESR peak at *g*=*g*_t_ and *θ*=90° in 17 nm Ge QWs, as a function of the microwave power (*P*). The scale bar indicates the ESR signal intensity. *H*_1_ is the intensity of the magnetic field of the microwave within the sample. Inset: 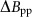 of the CESR peaks versus *P* (dots) and fit (solid line) according to ref. 56. The error bars are derived from the least squares fit analysis of the lineshape of the spectrum. (**b**) Peak-to-peak amplitude (*A*_pp_) of the peaks shown in **a** versus *P*. The blue and orange solid lines are guides to the eye. The shape of the peak at −30, −12.5 and −7 dB are reported (black lines) together with the fitting curves (coloured lines) showing absorption (AS), dispersion (DS) and polarization (PS) components^56^, respectively. *A*_pp_ is considered positive when the ESR peak is AS-like, and negative when it is PS-like. The error bars are derived from the least squares fit analysis of the lineshape of the spectrum. (**c**) Values of spin-lattice relaxation time *T*_1_ and ensemble dephasing time 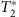 obtained from ESR peaks at *g*=*g*_t_ in QWs with different thickness. The error bars are derived from the least squares fitting analysis based on the model of ref. 56 of the data set measured at different microwave power.

Figure 5a shows a colour-coded map of the CESR intensity as a function of *P* in the −30 dB (low *P*) to −7 dB (high *P*) range for the resonance peak corresponding to degenerate C and D valleys measured at *θ*=90° in the sample with 17 nm thick QWs. Figure 5b shows that at low *P* the CESR signal possesses the well-known absorption lineshape (AS), which results from spin–flip processes induced by the resonance between the microwave photons and the Zeeman splitting of the spin states. For a direct inspection, the CESR peak measured at −30 dB is shown as a black line in the inset in Fig. 5b. The typical increase and saturation with *P* of the AS lineshape, which is routinely observed in electron spin resonance experiments^54^, might not be easily seen in our data (inset of Fig. 5a). Nevertheless, a puzzling behaviour can be appreciated in Fig. 5. At low *P*, the lineshape resembles the well-studied Dysonian shape observed in metals when dispersion of the microwave power arises because of skin effects at the metal surface^54^. The pattern is asymmetric because of the occurrence of an additional dispersion signal (DS), which in 2DEGs was reported for the first time in Si QWs and explained by considering the real component of the magnetic susceptibility of the samples^56^. Indeed, by increasing *P* at first the AS becomes weak and at *P*∼−12 dB the lineshape gets fully modified, showing one unexpected negative dip, which stems from a pure DS (see also inset of Fig. 5b). Notably, by further increasing *P* the intensity of the resonance peak turns out to be strongly enhanced and the lineshape changes again showing this time a parity inversion with respect to the AS-like pattern of the low power regime (see also inset of Fig. 5b). Such sign change of the absorption component compares well with the polarization signal (PS) occurring in 2D conduction electrons because of variations of the spin-dependent conductivity during the microwave absorption process^56^.

In light of this discussion, the overall behaviour of the CESR lineshape as a function of *P* can be accounted for by a linear superposition of the three AS, DS and PS contributions (see Supplementary Note 5). According to the model put forward in ref. 56, the latter leads to a peak-to-peak amplitude *A*_pp_ that scales as 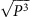, while AS and DS are both proportional to 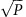. Figure 5b, where we assumed negative amplitudes for PS-like peaks, shows that such a phenomenological power law well describes our findings as AS (PS) dominates at low (high) *P*, while AS and PS cancel each other in the intermediate regime, eventually making the DS component clearly visible at *P*∼−12 dB.

As detailed in Supplementary Note 5, modelling the resonance lines by these three signal components provides us with the *T*_1_ and 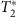 times summarized in Fig. 5c for all the QW samples. For the sample with the thickest QWs, the model gives 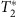 in good agreement with those anticipated in Fig. 4b for all *g* factors, further corroborating our previous linewidth analysis. Figure 5c also shows that 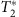 decreases and its values at 90° and 35° get closer in thinner QWs. This behaviour compares well with an enhancement in the electron localization when the QW width is reduced, and with the correspondingly increasing efficiency in the spin dephasing due to hyperfine coupling. Above all, Fig. 5c discloses *T*_1_ values in the μs regime, thus more than two orders of magnitude longer than 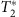. The accuracy of these findings is given by the analysis of the CESR linewidth reported in Fig. 5a for the 17 nm thick QWs. The modelling of the linewidth, as described in ref. 56, is shown as a solid line in the inset of Fig. 5a and provides a *T*_1_=1 μs, in good agreement with the result of the CESR-amplitude study shown in Fig. 5c.

It is worth noting that spin-lattice relaxation times derived in our QWs approach 5 μs and are substantially longer than the one reported for conduction electrons in bulk Ge at the same temperatures (see Table 2). While *T*_1_ below 1 μs were experimentally obtained in bulk Ge between 30 and 60 K (ref. 21), in satisfactory agreement with the Elliott–Yafet prediction for thermal electrons^20^, various attempts failed to recover such consistency at lower temperatures (see ref. 29 and refs. therein). In this regime, theory suggests a *T*_1_ extending well above the μs range, whilst an experimentally attainable upper bound of ∼100 ns was notably singled out at 4 K (ref. 29). All these endeavours put forward the subtle role played by impurities in introducing extrinsic spin-relaxation channels^59^,^60^ that in bulk Ge emerge at low temperatures and drastically prevail over the intrinsic but slower Elliott–Yafet process. By spatially separating conduction band electrons residing in the Ge QW from their parent donor atoms embedded in the SiGe barriers, we prevent the impurity-induced bottleneck pertaining to experiments utilizing bulk Ge wafers, and eventually resolve long-lived spins despite the low temperature operation.

In the Orbach-dominated regime, donor-bound electrons have been shown to retain in Ge exceedingly long *T*_1_ values approaching 100–300 μs (ref. 28). Such findings demonstrate that going from itinerant to immobile, fully-localized electrons, while inducing hyperfine dephasing, can be also beneficial in quenching the spin-lattice relaxation activated by impurities and Elliott–Yafet spin–flip. This suggests that quantum confinement, guaranteed in our Ge/SiGe heterojunctions by the type I band alignment, possibly provides an additional mechanism concurring to the lengthening of *T*_1_ that arises when the conduction electrons reside in QWs rather than in bulk material.

### Optical measurement of *T*_1_

To gather a deeper understanding of the spin physics offered by the Ge QWs and to substantiate further the spin-lattice relaxation times inferred from CESR data, we carried out time- and polarization-resolved photoluminescence (PL) measurements (see ‘Methods' section).

In this case, we leverage spin–orbit to achieve optical spin orientation through absorption of photons carrying angular momentum. The selection rules for electric dipole interband transitions with circularly polarized light allow the excitation of a non-equilibrium population of carriers in the vicinity of the Γ-point having a net spin orientation along the propagation direction of the optical beam.

In Ge QWs, the energy relaxation of the photoexcited holes, towards the centre of the Brillouin zone in the valence band, is accompanied by a quick spin depolarization occurring in a sub ps regime^61^,^62^. On the other hand, ultrafast scattering events deplete the optically excited conduction band levels on a hundreds-of-fs time scale^61^,^62^. Electrons transferred out of Γ will eventually accumulate at the bottom of the L-valley^63^, where they reside for a few ns^64^. The electron spin polarization is notably not extinguished during such non-trivial processes. Finally, it will govern the radiative recombination with the unpolarized hole-pocket at Γ, yielding circularly polarized PL^61^.

The spectral resolution of the optical transitions therefore offers us the possibility to selectively access the spin dynamics of L-valley electrons, by observing the time-decay of the polarized indirect PL emission.

**Figure 6:**
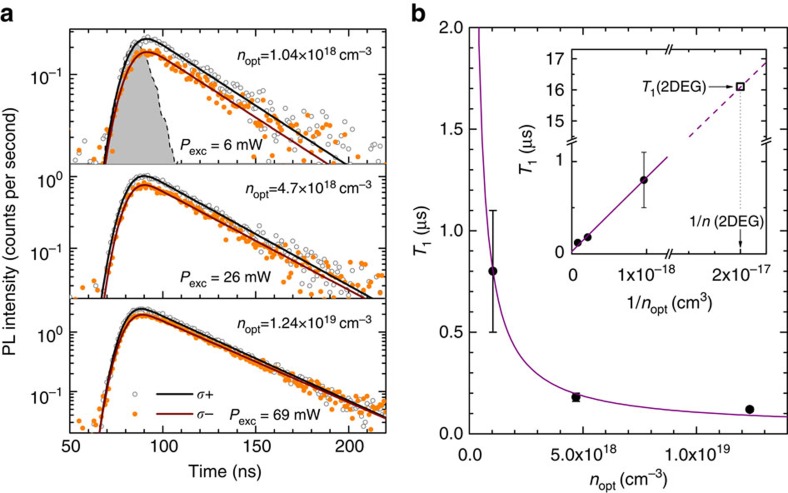
Estimation of *T*_1_ from time and polarization resolved PL. (**a**) Decay curves of the indirect PL emission from a 20 nm thick QW. The filled area corresponds to the laser pulse. Co-circular (*σ*+) and counter-circular (*σ*−) emissions with respect to the excitation have been obtained at *T*=4 K, under excitation by 1.165 eV circularly polarized laser line. The three panels report data (dots) and fits (lines) of the PL for the various densities of the photogenerated carriers *n*_opt_. (**b**) *T*_1_ values (dots) obtained by using the PL data in **a**. The solid line is a fit of *T*_1_ as a function of 1/*n*_opt_. The inset shows the *T*_*1*_(2DEG) obtained by the fit for the electron concentration *n*(2DEG) pertaining to the n-mod QW studied by CESR.

Figure 6a reports the intensity decay versus time of the no-phonon line of the indirect PL emission. The PL was measured at 4 K in 20 nm thick undoped Ge QWs under excitation by circularly polarized light at 1.165 eV. This pump energy excites carriers directly in the Ge QWs, due to the negligible absorption occurring in the SiGe barrier layers. In Fig. 6a, the co-circular (*σ*+, grey open dots) and counter-circular (*σ*−, orange full dots) emissions with respect to the right handed *σ*+ excitation are reported for different pump powers, while the black (brown) solid lines are the fits of the *σ*+ (*σ*−) decay curves.

The different intensities of the two helicity-resolved PL components at the early stage of the recombination dynamics demonstrate a net circular polarization of the emission. This is indeed the clear signature of the successful optical spin orientation of L-valley electrons. It is worth noting that the time-decay of the optically-induced non-equilibrium population of the electron spins reflects itself by the time-dependent depolarization of the PL. The latter, in turns, provides a direct means to measure the spin-lattice relaxation time^65^.

Figure 6a demonstrates that by increasing the pump power, the initial PL polarization decreases. Moreover, the equalization of the PL intensity of the right- and left-handed components occurs at earlier times, implying shorter *T*_1_ values. Surprisingly, the spin-lattice relaxation times corresponding to these excitation regimes (dots in Fig. 6b) turn out to be systematically shorter than those found by CESR in the n-mod QW counterpart (Fig. 5c).

The puzzling shortening of *T*_1_ observed in the PL experiments unveils additional relaxation mechanisms that by far outweigh the intrinsic Elliott–Yafet process and the otherwise dominant impurity-driven relaxation, enriching further the intriguing spin dynamics in the Ge QWs.

Figure 6b shows a monotonic dependence of *T*_1_ on the density (*n*_opt_) of electrons optically injected in the QWs at the various excitation power levels (see Supplementary Note 6). Crucially, the density of photoexcited carriers turns out to be much larger than the one introduced by the remote doping in the n-mod samples studied by CESR, that is, 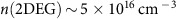 (see Supplementary Note 6). The well-defined density-dependent characteristics and the marked suppression of *T*_1_ shown in Fig. 6b openly manifest the surge of the electron-hole exchange interaction^66^,^67^. Possibly, this spin-relaxation channel is strengthened in the QWs by the spatial confinement of the carriers. This might have concealed its direct observation in previous literature reports dealing with bulk Ge (refs 29, 58).

After having discussed the spin-relaxation mechanisms in the optically-pumped Ge QWs, we can attempt to reconcile PL and CESR data even though the excessively weak PL intensity precludes the measurement at 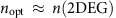. Since, to a first approximation, the *T*_1_ dependence on *n*_opt_ can be modelled as 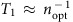 (solid line in Fig. 6b), we can disentangle the exchange interaction from the optically derived data and extrapolate the spin-lattice relaxation pertaining to the n-mod Ge QWs studied by CESR. Following this line of reasoning, a *T*_1_ value of 16 μs is found for the 2DEG (inset Fig. 6b), in good agreement with spin-lattice relaxation times inferred from CESR measurements (Fig. 5c). These findings, in spite of the simplified approach, corroborate the CESR analysis and provide central insight into the rich spin dynamics occurring in Ge QWs.

## Discussion

The spin properties of conduction electrons in Ge QWs can be investigated independent of donors and strain only in well-designed heterostructures. Our work based on n-mod Ge/SiGe QWs provides direct access to intrinsic spin-dependent phenomena and demonstrates the potential of Ge in enriching group IV spintronics and enabling quantum technologies. Our findings point out that the 2DEG can be surprisingly accompanied by a *g*-tensor mimicking the one of bulk material, a result that might stimulate further experimental and theoretical investigations.

Inspired by a recent experimental report on donor-bound electrons^68^, we can foresee that the demonstrated strong dependence of the electron Landé *g* factor upon confinement can be utilized in conjunction with externally applied electric fields to provide an exceptional tunability . With this respect, the anisotropy may be additionally fine-tuned by a Rashba field induced by asymmetric doping and subsequently modulated via an external gate^27^.

CESR and PL studies unveiled microsecond long spin-lattice relaxation times in the low temperature regime, which strikingly match the *T*_1_ values measured in Si QWs (refs 25, 69). Such result sheds light on the coexistence of long spin-relaxation times and large *g* factor variations, which support Ge as an excellent candidate for the exploitation of spin currents in novel transport architectures, such as spin-based interconnects^70^, transistors^71^ and reprogrammable logic^72^.

We notice that strain engineering and heteroepitaxy on (111)-oriented substrates have been recently put forward by theoretical studies^19^,^20^,^59^ as a means to lift the valley degeneracy. Such an approach can be effectively utilized to further lengthen the spin-lattice relaxation times and extend our results to higher temperatures, in a regime where intervalley scattering provides the major spin-loss mechanism.

Eventually, by uniquely combining CESR and PL, we were able to demonstrate the existence of exchange-driven relaxation mechanisms that markedly depend upon the non-equilibrium carrier density.

Looking ahead, 2DEGs in Ge can offer a special framework for quantum computation, in particular electrostatically defined Ge quantum structures on Si can open unexplored pathways for future studies of confinement-induced tailoring of the spin physics in group IV semiconductors.

## Methods

### Sample growth

Ge/SiGe QWs were grown by low energy plasma enhanced chemical vapour deposition^73^ at 475 °C on p-Si(001) substrates with a resistivity of 1–10 Ω cm. Before heteroepitaxy, RCA cleaning was carried out, and the native oxide was removed by dipping the substrate in HF solution (HF:H_2_O 1:10) for 30 s. The first part of the structure consists of a 13 μm thick Si_1*−x*_Ge_*x*_ graded buffer, deposited at a rate of 5–10 nm s^−1^, in which the Ge concentration is linearly increased from 0 to 92.5%. A 2 μm thick relaxed Si_0.075_Ge_0.925_ layer was deposited on top of the graded buffer. Finally, the stack of 500 pure Ge QWs embedded in 21 nm thick Si_0.15_Ge_0.85_ barriers was deposited with a rate of 5 nm s^−1^. Due to plasma confinement, the growth rate is not uniform across the 4″ wafer. For this reason samples with different QW width but exactly the same stoichiometry of the barrier layer and comparable strain are provided in one growth run. In the middle of each barrier, co-deposition of P was used to provide a 10 nm layer with P concentration of 10^11^ cm^−2^, yielding n-type modulation doping of the QWs. Finally, a 40 nm thick Si_0.075_Ge_0.925_ layer and a relaxed crystalline Si capping layer having 10 nm of thickness were also deposited.

### X-ray diffraction

High-resolution x-ray diffraction measurements were performed using a PANalytical X'Pert PRO MRD diffractometer: The system is equipped with a hybrid mirror and a two-bounce asymmetric Ge monochromator for a high-intensity Cu K_α1_ beam. The beam size in this configuration is 2 mm × 20 mm. Reciprocal space maps were taken around the (004) and (224) Bragg reflections. The average Ge content and strain are obtained from the position of the 0th-order peak in the reciprocal space. The period of the superlattices is calculated from the satellite peaks period. Composition and thickness of QW and barrier layers are extracted by the intensity profile of the satellites along the out-of-plane component of the scattering vector *Q*_z_.

### Electronic structure calculation

The band alignment and electronic wave functions of the remotely doped QW structures were calculated within the effective mass approximation by using a Schrödinger–Poisson solver implemented in Nextnano^74^. The set of deformation potentials used for the calculations is reported in ref. 75 and the average valence band offset between Si and Ge was chosen to be 800 meV according to ref. 76.

### Atomic force microscopy

We used a Veeco Innova atomic force microscopy (AFM). The microscope was used in tapping mode imaging. We made images of 10 μm × 10 μm area or larger, using a ultra-sharp tip. Samples were rinsed in acetone and isopropanol before carrying out the measurements. AFM images of the surface of the samples provided a r.m.s. roughness between 1.7±0.2 nm and 2.2±0.2 nm.

### Electron spin resonance

Two continuous wave EPR spectrometers were employed: A Bruker Elexsys E500 with Bruker ER4102ST rectangular cavity and a Varian E-9 magnet with E-101 microwave bridge (X-band, ∼9.5 GHz) with super-high Q ER4122SHQE cylindrical cavity. Oxford ESR910/ESR900 Liquid He cryostat operating below 2 and 4 K were used with the two spectrometers, respectively.

### Time- and polarization-resolved photoluminescence

PL experiments were performed in backscattering geometry using a Nd:YAG Q-switched laser at 10 kHz frequency, whose pulses have a temporal width of about 10 ns. The circularly polarized laser light was focused to a 53 μm diameter spot, and the emission was probed by a photomultiplier tube (Hamamatsu R5509–73) coupled to a monochromator. The band pass was 3.55 nm, and the time resolution of the whole detection system 5 ns. The sample was mounted in a cold finger closed-cycle cryostat.

### Data availability

The data that support the findings of this study are available from the corresponding authors on request.

## Additional information

**How to cite this article:** Giorgioni, A. *et al*. Strong confinement-induced engineering of the *g* factor and lifetime of conduction electron spins in Ge quantum wells. *Nat. Commun.* **7,** 13886 doi: 10.1038/ncomms13886 (2016).

**Publisher's note:** Springer Nature remains neutral with regard to jurisdictional claims in published maps and institutional affiliations.

## Supplementary Material

Supplementary Information Supplementary Figures 1-4, Supplementary Notes 1-6 and Supplementary References.

## Figures and Tables

**Table 1:** Electron *g* factor in Si–Ge materials systems.

**Reference**	**Sample**	***g*** **factor**	**Δ*****g*** **tuning range**	**Tuning mechanism**	**Electrons**	**Study**
31	intrinsic Ge	0.9–2.04			3D	Theory
45	Ge:Sb	0.820–1.922			Bound (strained)	Exp.
46	Ge:Sb	1.56			Bound	Exp.
43	Ge:P	1.5631			Bound	Exp.
43	Ge:As	1.570			Bound	Exp.
27	Si QW	1.9944	10^−3^ (*g*=1.9944)	Electric current	2D	Exp.
53	SiGe QDs	1.9992–1.9994	2 × 10^−4^ (*g*=1.9992)	Confinement	0D	Exp.
52	SiGe QDs	1.9992	7 × 10^−4^ (*g*=1.9992)	Confinement	0D	Exp.
42	Ge QWs	0.82–1.93	0.010 (*g*=1.325)	Confinement	2D	Theory
68	Ge:P	1.5631	10^−6^–7 × 10^−4^ (*g*=1.5631)*	Electric field	Bound	Exp.
This work	Ge QWs	0.905–1.915	0.009 (*g*=1.328)	Confinement	2D	Exp.

exp, experiment; QW, quantum wells. QD, quantum dots.

Experimental and theoretical reports of the electron *g*-factor values of prominent group IV materials and related mechanisms exploited for its manipulation. In the third column a single value stems from isotropic *g* factors, while extremal values are reported for the anisotropic case.

^*^Lowest value measured at 25 V cm^−1^, and highest value extrapolated to 480 V cm^−1^.

**Table 2:** Spin relaxation and dephasing times for electrons in Ge.

**Reference**	**Sample**	***T***_**1**_ **(μs)**	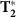 **(ns)**	***T*** **(K)**	**Electrons**	**Technique**
21	Intrinsic Ge	0.2–0.9		30–60	Cond. - 3D	Hot-electron transport
58	Intrinsic Ge	0.017–0.065	26–36	10–50	Cond. - 3D	Faraday rotation
29	Intrinsic Ge	0.127–0.26		4–50	Cond. - 3D	Photoluminescence
25	Si QWs	2–3	1,400	4–5	Cond. - 2D	cw and pulsed ESR
This work	Ge QWs	1–5	10–30	2–5	Cond. - 2D	cw ESR and PL
28	7.8% ^73^Ge:As	50–800	11	2–5	Bound	Pulsed ESR
28	0.1% ^73^Ge:P	10–500	211	0.3–5	Bound	Pulsed ESR

cond, conduction; ESR, electron spin resonance; exp, experiment; cw, continuous wave; PL, photoluminescence; QW, quantum wells.

Longitudinal relaxation *T*_1_ and decoherence time of the spin ensemble 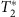 of conduction (cond.) and bound to donor electrons. The samples, the temperature range (*T*) and the techniques utilized to experimentally determine the values are also indicated.
