# Supplementary material for: Strong confinement-induced engineering of the g factor and lifetime of conduction electron spins in Ge quantum wells
Source: Nat Commun. 2016 Dec 21;7:13886. doi: 10.1038/ncomms13886 (PMC5187588; doi:10.1038/ncomms13886)
Supplement: Supplementary Information — Supplementary Figures 1-4, Supplementary Notes 1-6 and Supplementary References. [file ncomms13886-s1.pdf]

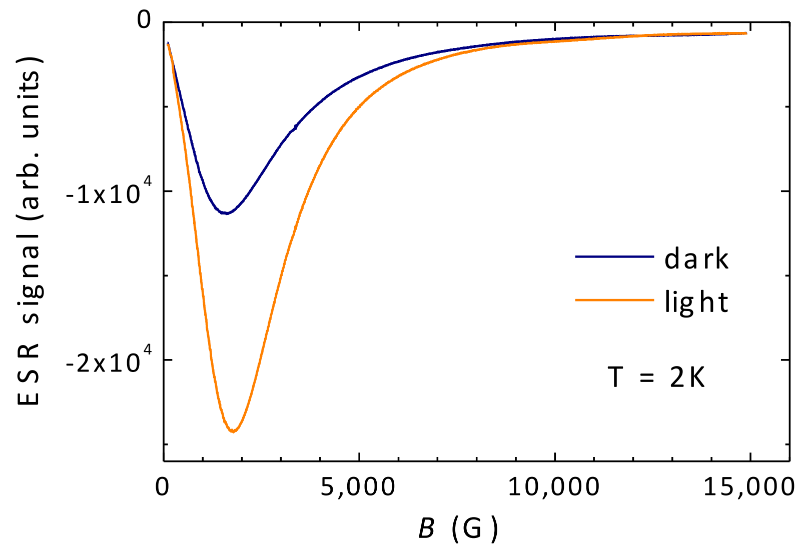

Supplementary Figure 1. Cyclotron Resonance (CR) and evidence of free carriers in Ge QWs at low temperature and B//[001]. The signal intensity increases under illumination with a 532 nm laser. The energy of this laser is well above the optical gap of the QWs [1]. Since the integrated intensity of the CR signal is proportional to the number of carriers [2], and free carriers are photogenerated by light absorption [1], the figure indicates that the CR signal stems from free carriers.

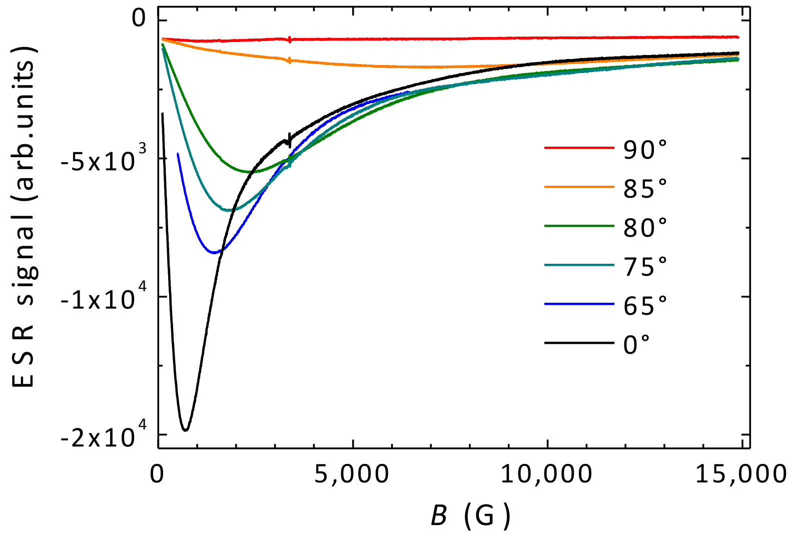

Supplementary Figure 2. Angular dependence of the CR. The CR signal decreases as $\theta \rightarrow 0$ according to $1/\cos\theta$, which is a signature of the 2DEG [3]

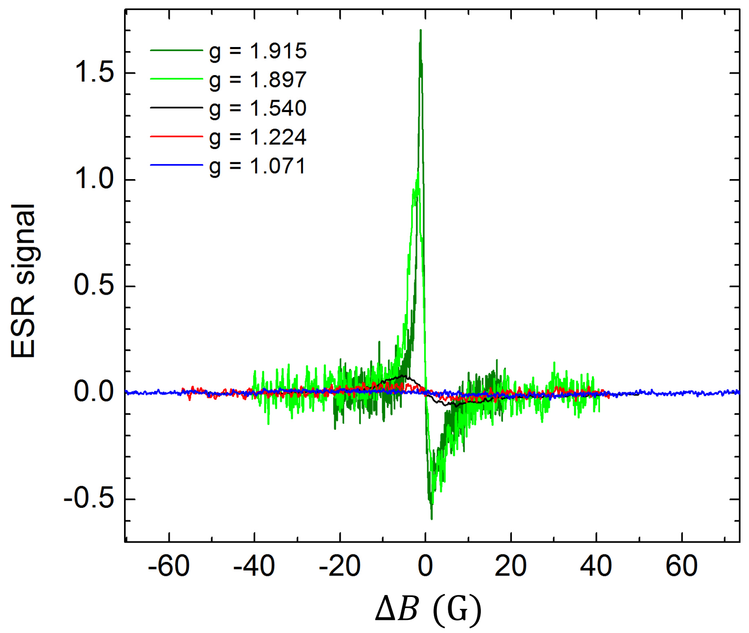

Supplementary Figure 3. Conduction Electron Spin Resonance (CESR) signal intensity vs g factor. Raw data as a function of $\Delta B = B - B_{\mathrm{res}}$, where B_{res} is the resonance field. ESR in Ge QWs presents a highly anisotropic g factor and linewidth. In particular, the linewidth increases when the g factor decreases. Since the area of the ESR signal is proportional to the number of contributing spins, the increase in the linewidth is always accompanied by a reduction of the signal intensity. In the figure, this effect is highlighted by showing the CESR spectra in 20 nm width Ge QWs. Significantly lower intensities prevented the observation of CESR signals when $g < 1.05$.

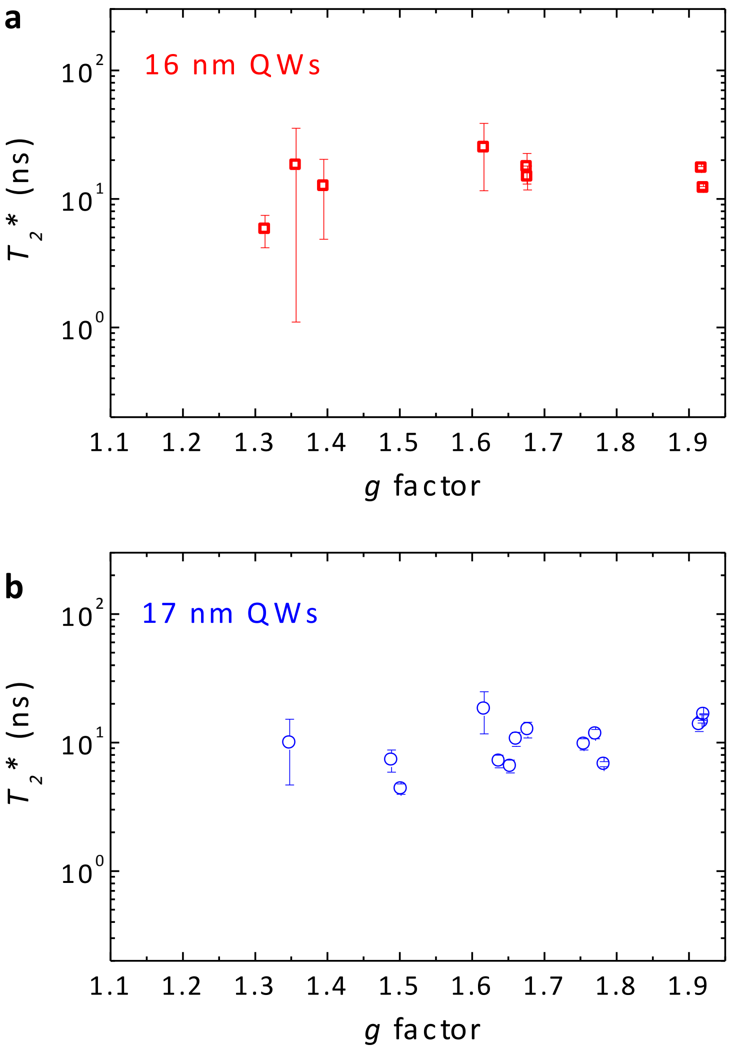

Supplementary Figure 4. Estimation of T_2^* for inhomogeneous broadening of the line due to QWs width fluctuation. a, b T_2^* values obtained in 16 nm and 17 nm QWs, respectively. For each sample the calculated $\Delta B^{\mathrm{G}}{}_{\mathrm{pp}}(g)$ was used to estimate ΔB_{pp} through Equation (3), and successively T_2^* by Equation (2). Times are found in the order of 10 - 20 ns in both samples. Error bars were derived from the linewidth uncertainties. For further details see Supplementary Note 3.

Supplementary Note 1. Structural parameters of the Ge QWs. Each sample consists of a 500-fold stack of Ge/$Si_{0.15}Ge_{0.85}$ multiple QWs. HRXRD measurements allowed us to estimate the QW width (L_z), the strain level of the layers, and the final composition of the buffer. The values measured for L_z are 20 ± 1 nm, 17 ± 1 nm and 16 ± 1 nm. All the samples features an identical in-plane strain in the QW region equal to $\varepsilon_{\|} = -2.2 \cdot 10^{-3}$. In all cases the graded buffer final composition is found to be $Si_{0.07}Ge_{0.93}$ with a residual in-plane strain $\varepsilon_{\text{buffer}} = 10^{-3}$. Such tensile strain is built up during cool down after the epitaxial deposition because of the thermal expansion coefficient mismatch between the constant composition layer of the graded buffer and the silicon substrate, and results in a corresponding reduction of the compressive strain in QW layers.

Supplementary Note 2. CESR *g* factor vs θ. When **B** is rotated in the (1-10) plane, from the [001] to the [110] crystallographic direction, the electrons belonging to the four *L* valleys have the following *g* factors[4]:

$$g_{\mathrm{A}}{}^2 = g_{\mathrm{p}}^2 \cos^2(\theta + \theta_0) + g_{\mathrm{t}}^2 \sin^2(\theta + \theta_0) \qquad (1)$$

$$g_{\mathrm{B}}{}^2 = g_{\mathrm{p}}^2 \cos^2(\theta - \theta_0) + g_{\mathrm{t}}^2 \sin^2(\theta - \theta_0) \qquad (2)$$

$$g_{\mathrm{C,D}}{}^2 = \frac{1}{3} g_{\mathrm{p}}^2 \cos^2\theta + g_{\mathrm{t}}^2 (1 - \frac{1}{3}\cos^2\theta) \qquad (3)$$

where θ is the angle between **B** and the normal to the sample surface, the indices g_i refer to the four branches, and $\theta_0 = \operatorname{acos}\frac{1}{\sqrt{3}}$.

Supplementary Note 3. Inhomogeneous broadening of the ESR line due to QW width fluctuations, and estimation of T_2*. Since the g factor depends on the QW width, rough interfaces between QW and the barrier layers will cause inhomogeneous broadening of the ESR lines. An estimation of the roughness is obtained measuring the Root-Mean-Square (RMS) roughness of the sample surface by Atomic Force Microscopy (AFM). The main text reports an AFM image of the sample with L_z = 20 nm thick QWs in Fig.4b, which yields a RMS roughness $\Delta L_z = 2.2 \pm 0.2$ nm. We assume a value of 20 nm as the average L_z, and a Gaussian distribution of the well widths having a σ of $\Delta L_z/2 = 1.1 \pm 0.1$ nm. The value of g_t is shown to be independent of L_z. The value of g_p instead depends on L_z. The measured data, assuming a linear dependence in the vicinity of the L_z = 20 nm point, yield:

$$\frac{\partial g_{\mathrm{p}}}{\partial L_{\mathrm{z}}} = 0.0035\ \mathrm{nm}^{-1} \qquad (4)$$

A Gaussian distribution of g factors, together with the ellipsoidal anisotropy of g result in an inhomogeneous broadening $\Delta B^{\mathrm{G}}{}_{\mathrm{pp}}$, which depends on the g value, as described by the relations[5]:

$$\Delta B^{\mathrm{G}}{}_{\mathrm{pp}}(g) = \frac{h\nu}{\mu_B}\frac{1}{g^2}\,\Delta g\ ; \qquad \Delta g = \frac{g_{\mathrm{t}}-g}{g_{\mathrm{t}}-g_{\mathrm{p}}}\,\sigma_g \qquad (5)$$

where $h\nu$ is the microwave energy, μ_{B} the Bohr magneton, and σ_g in Ge QWs is given by:

$$\sigma_g = \frac{\partial g_{\mathrm{p}}}{\partial L_{\mathrm{z}}} \cdot \Delta L_{\mathrm{z}} = 0.0035\ \mathrm{nm}^{-1} \cdot (2.2 \pm 0.2\ \mathrm{nm}) = (7.7 \pm 0.8) \cdot 10^{-3} \qquad (6)$$

$\Delta B^{\mathrm{G}}{}_{\mathrm{pp}}(g)$ was calculated by means of Supplementary Equation 5 for 20 nm QWs. The uncertainties were derived from the RMS of the roughness alone, since the uncertainties on g_{p} and g_{t} are far smaller.

The same analysis was carried out in 16 nm and 17 nm QWs, yielding similar results. It is worth noting that different coefficients were employed, namely $\frac{\partial g_{\mathrm{p}}}{\partial L_{\mathrm{z}}} = 0.006$ in 16 nm wells, and $\frac{\partial g_{\mathrm{p}}}{\partial L_{\mathrm{z}}} = 0.004$ in 17 nm wells, due to the dependence of g_{p} upon L_{z}.

Supplementary Note 4. Spin dephasing mechanisms in Ge QWs. In the following we list the expected processes that can contribute to spin dephasing of free electrons in Ge QWs, and we indicate their effects on the linewidth of the ESR lines.

#1: Intervalley spin-conserving scattering. In Ge-based QWs there are four L-valleys with their main axes oriented along the {111} directions. Therefore, the g-factors tensor is diagonal when the magnetic field lies along one of these directions, and it is non-diagonal for the other field orientations. In the presence of intervalley scattering between the valleys with different orientations of the main valley axes with respect to **B**, spin dephasing takes place at spin-independent scattering and is given by[6]:

$$\tau_{\mathrm{s}} = \frac{\tau}{1-|\cos\theta|} \qquad (7)$$

where θ is an angle between the directions of the Larmor precession frequency vectors in the two valleys, $\Omega_{\mathrm{L}}{}^{(1,2)}$, and τ is the intervalley scattering time.

Supplementary Eq.7 holds when $\Omega_{\mathrm{L}}\tau \gg 1$. If on the other hand $\Omega_{\mathrm{L}}\tau \ll 1$, then the intervalley scattering does not depend on θ and we have:

$$\frac{1}{\tau_{\mathrm{s}}} \approx \left|\Omega_{\mathrm{L}}{}^{(1)} - \Omega_{\mathrm{L}}{}^{(2)}\right|^2 \tau \quad (8)$$

In our experiment Ω_{L} is given by the microwave frequency, and to a first approximation the intervalley scattering time τ will be about 200 fs [7]. As a result, $\Omega_{\mathrm{L}}\tau \sim 5.9 \cdot 10^{10}\mathrm{Hz} \times 200 \cdot 10^{-15}\mathrm{s} \sim 1.2 \cdot 10^{-2} \ll 1$. This defines the regime where this dephasing mechanism is anisotropic. It shall be noted that we found an isotropic T_2^*.

#2: Intervalley spin-flip scattering. These processes also lead to spin relaxation, as discussed in detail in the paper by Liu *et al.* [8].

#3: Elliott-Yafet spin relaxation. Due to the spin dependence of electron scattering, the scattering probability from $\mathbf{k}$ to $\mathbf{k}'$ has a term $W_{\mathbf{k}'\mathbf{k}} \propto \boldsymbol{\sigma} \cdot (\mathbf{k}\times\mathbf{k}')$. This term is present in any semiconductor irrespectively of the presence or absence of an inversion centre. For 2D electrons, this term results in relaxation of spin components in the 2D plane and does not affect the out-of-plane spin component:

$$\tau_{\mathrm{s,xx}} = \tau_{\mathrm{s,yy}} = \tau_s \quad \tau_{\mathrm{s,z}} = \infty \quad (9)$$

As a result, for the geometry $\boldsymbol{B} \parallel \mathrm{z}$, we get $T_2 = \tau_{\mathrm{s}}$, while for geometry $\boldsymbol{B} \perp \mathrm{z}$, the result is $T_2 = 2\tau_{\mathrm{s}}$. Our experimental data are consistent with this anisotropy induced by the Elliott-Yafet spin relaxation. In reality, the spin-flip scattering probability has a more complicated form with a few linearly-independent constants due to the low symmetry $C_{3\mathrm{v}}$ of the valley. Taking this fact into account can yield an anisotropy factor different from 2.

Supplementary Note 5. Lineshape model. At $g = g_{\mathrm{t}}$, the Gaussian broadening due to interface roughness vanishes. Therefore the ESR line of conduction electrons is fully described by the AS, DS, and PS contributions introduced in the main text.

#1: absorption signal (AS). It originates from the interaction of the sample with the magnetic field of the microwave through the imaginary part of the magnetic susceptibility. It is given by the spin-flip microwave absorption due to magnetic-dipole transitions, and is well described by:

$$f'_{\mathrm{AS}} = -\frac{1}{\pi}\frac{2h}{(1+p+h^2)^2} \quad (10)$$

where $h = \sqrt{3}\,(B - B_0)/\Delta B^{\mathrm{L}}_{\mathrm{pp}}$, with B_0 the resonance field, and $\Delta B^{\mathrm{L}}_{\mathrm{pp}}$ the Lorentzian linewidth. $p = \gamma^2 H_1^2 T_1/\,\Delta\omega$ with γ the gyroscopic factor, H_1 the amplitude of the magnetic field of the microwave on the sample, T_1 the spin-lattice relaxation time, and $\Delta\omega$ the linewidth of the ESR line. p is proportional to the microwave power due to the term H_1^2. The low power linewidth (far from saturation) is related to the spin relaxation rates by the following[9]:

$$\Delta\omega = \frac{1}{2T_1} + \frac{1}{T_2} \qquad (11)$$

#2: dispersion signal (DS). It is provided by the interaction of the sample with the electric field of the microwaves through the imaginary part of the electric conductivity, in the case of high mobility electrons. It is described by:

$$f'_{\mathrm{DS}} = \frac{1}{\pi}\frac{1+p-h^2}{(1+p+h^2)^2} \qquad (12)$$

#3: polarization signal (PS). It is due to the interaction of the sample with the electric field of the microwave. This signal arises when the electric conductivity of the sample depends on the spin polarization of the carriers. Indeed, when this condition is verified, the electrical response of the sample changes during the resonant microwave absorption, because the spin population changes at resonance. PS was already observed in Si QWs[10], and it has the following shape:

$$f'_{\mathrm{PS}} = -\frac{2ph(1+h^2)}{(1+p+h^2)^3} \qquad (13)$$

These three contributions sum up in the shape function S' describing the ESR line measured at $g = g_{\mathrm{t}}$. We observed that at fixed QW width and θ, S' depends on the microwave power P (see Fig.5a in the main text). Particularly, the lineshape depends on P, while P does not affect significantly ΔB_{pp}, which is related to $\Delta\omega$. As discussed in the study of the linewidth, the measurement of T_2 is limited by broadening factors, thus we can obtain a lower limit of T_2, called $T_2{}^*$. It is not trivial to extract time estimations from the lineshape, because T_1 and T_2 are coupled in the shape functions of the three contributions (Supplementary Eqs. 10, 12, and 13). However, since T_1 and T_2 are related through the $\Delta\omega$ expression in Supplementary Eq. 11, a lower-limit estimation of T_2 gives us the possibility to get an upper-limit of T_1. The overall shape function S' has the following form:

$$S' = A_{\mathrm{AS}} \cdot f'_{\mathrm{AS}} + A_{\mathrm{DS}} \cdot f'_{\mathrm{DS}} + A_{\mathrm{PS}} \cdot f'_{\mathrm{PS}} \qquad (14)$$

where A_{AS}, A_{DS}, and A_{PS} are the weights of the contributions, independent of P; f'_{AS}, f'_{DS}, and f'_{PS} instead depend on P through the relaxation times. For every QW width, the whole set of ESR peaks measured as a function of P (at $g = g_{\mathrm{t}}$ and fixed θ) can be fitted by Supplementary Eq. 14, yielding the values of T_1 and T_2^* (reported in Fig. 5c in the main text), and the appropriate values for A_{AS}, A_{DS}, and A_{PS}.

Supplementary Note 6. Extrinsic and photogenerated carrier concentration in Ge QWs. In order to compare the T_1 values obtained by ESR and optical measurements, we consider the electron concentration in the two techniques, in 20 nm QW samples. ESR measurements were carried out on n-mod doped samples, at T in the range 2 - 4.5 K. At room temperature, nearly all P atoms are ionized due to the thermal energy[11], and according to the potential profile (Fig. 1d in the main text) the donor electrons occupy the lowest energy states of the conduction band in the QW layers. As a consequence, also at cryogenic temperatures donor electrons reside in the QWs, in agreement with the absence of ESR signals from P donors. Since the sheet density of P atoms in each barrier is $\sim 10^{11}\mathrm{cm}^{-2}$ and the QW width 20 nm, than the concentration of extrinsic electrons in each QW is $n(\mathrm{2DEG}) \sim 5 \cdot 10^{16}\mathrm{cm}^{-3}$.

In PL measurements of an undoped QW sample (carried out at T = 4 K) the concentration of photogenerated carriers depends on the excitation power level. Since the time resolved PL requires a pulsed source, we can calculate the number of carriers photoexcited by a laser pulse. The PL decay curves (Fig.6a in the main text) show that the carrier lifetime is ~ 30 ns, while the FWHM of the laser pulse is much shorter ($\Delta t < 14$ ns). As a result, we assume that no recombination takes place during the pulse absorption. For sake of simplicity, we model the laser pulses as rectangular wave form. The laser power was measured by a thermopile, providing the average P_{exc} between 6 and 69 mW, distributed on a laser spot size of 53 μm. The energy associated with a single pulse is $E_{\mathrm{pulse}} = P_{\mathrm{exc}}/f$, where f is the laser pulse frequency (10 kHz). The number of photons reaching the sample surface every pulse is thus $\phi_{\mathrm{pulse}} = E_{\mathrm{pulse}}/h\nu$, where $h\nu$ is the energy of a single photon, which has been chosen to be 1.165 eV, i.e. resonant with the direct gap transitions at the Γ point in the Ge QWs. Finally, the concentration of absorbed photons inside the sample depends on the surface reflectivity (R), and on the absorption coefficient (α) of the active material, according to the formula[12]:

$$n_{\mathrm{opt}} = \frac{\phi_{\mathrm{pulse}}\,(1-R)\alpha}{A} \qquad (15)$$

where A is the area of the laser spot at the sample surface. In the analysed samples $R = 0.38$. By using the literature data in the paper by Bonfanti *et al.* [1], we approximate $\alpha = 1.1 \cdot 10^4 \mathrm{cm}^{-1}$ for a single 20 nm thick QW. Carrier loss mechanism due to radiative recombination through the direct band gap can be neglected because the probability of electron transfer out of the Γ valley is about four order of magnitude larger than that of a radiative event at the zone center [13, 14]. Hence n_{opt} corresponds to the concentration of *L* valley electrons.

Supplementary References

1. Bonfanti, M. *et al.*, Optical transitions in Ge/SiGe multiple quantum wells with Ge-rich barriers. *Phys. Rev. B* **78**, 041407(R) (2008).

2. Palik, E. D. & Furdyna, J. K., Infrared and microwave magnetoplasma effects in semiconductors. *Rep. Prog. Phys.* **33** , 1193 (1970).

3. Wilamowski, Z., Jantsch, W., Malissa, H. & Roessler, U., Evidence and evaluation of the Bychkov-Rashba effect in SiGe/Si/SiGe quantum wells. *Phys. Rev. B* **66**, 195315 (2002).

4. Roth, L. M. & Lax, B., g FACTOR OF ELECTRONS IN GERMANIUM. *Phys. Rev.* **3**, 217-219 (1959).

5. Brower, K. L., Strain broadening of the dangling-bond resonance at the (111)Si-Si02 interface. *Phys. Rev. B* **33**, 4471-4478 (1986).

6. Glazov, M. M. *et al.*, Spin and valley dynamics of excitons in transition metal dichalcogenide monolayers. *Phys. Status Solidi B* **252**, 2349–2362 (2015).

7. Zhou, X. Q., van Driel, H. M. & Mak, G., Femtosecond kinetics of photoexcited carriers in germanium. *Phys. Rev. B* **50**, 5226-5230 (1994).

8. Liu, Z., Nestoklon, M. O., Cheng, J. L., Ivchenko, E. L. & Wu, M. W., Spin-Dependent Intravalley and Intervalley Electron-Phonon Scatterings in Germanium. *Phys. Solid State+* **55**, 1619 (2013).

9. Poole, C. P. J., *Electron Spin Resonance: a comprehensive treatise on experimental technique* (Dover Publications, 1996).

10. Wilamowski, Z. & Jantsch, W., Suppression of spin relaxation of conduction electrons by cyclotron motion. *Phys. Rev. B* **69**, 035328 (2004).

11. Xu, C., Senaratne, C. L., J., K. & Menéndez, J., Frustrated incomplete donor ionization in ultra-low resistivity germanium films. *Appl.Phys. Lett.* **105**, 232103 (2014).

12. Pavesi, L. & Guzzi, M., Photoluminescence of AlxGa1-xAs alloys. *J. Appl. Phys.* **75**, 4779-4842 (1994).

13. Pezzoli, F. *et al.*, Spin and energy relaxation in germanium studied by spin-polarized direct-gap photoluminescence. *Phys. Rev. B* **88**, 045204 (2013).

14. Lange, C. *et al.*, Ultrafast nonlinear optical response of photoexcited Ge/SiGe quantum wells: Evidence for a femtosecond transient population inversion. *Phys. Rev. B* **79**, 201306(R) (2009).
